# Exploring the effects of calycosin on anthracycline-induced cardiotoxicity: a network pharmacology, molecular docking, and experimental study

**DOI:** 10.3389/fcvm.2024.1286620

**Published:** 2024-03-21

**Authors:** Peng Zhu, Qianqian Ren, Ruizhi Zhang, Licai Zhang, Xiangwen Xia, Chuansheng Zheng, Tianhe Ye

**Affiliations:** ^1^ Department of Hepatobiliary Surgery, Wuhan No.1 Hospital, Wuhan, China; ^2^ Department of Radiology, Union Hospital, Tongji Medical College, Huazhong University of Science and Technology, Wuhan, China; ^3^ Hubei Province Key Laboratory of Molecular Imaging, Wuhan, China

**Keywords:** network pharmacology, molecular docking, anthracycline, cardiotoxicity, calycosin

## Abstract

**Background:**

Chemotherapy with anthracyclines can cause cardiotoxicity, possibly leading to stopping treatment in some cancer patients. In cardio-oncology research, preventing and minimizing anthracycline-induced cardiotoxicity (AIC) is a hot issue. For the treatment of AIC, calycosin (CA), an isoflavone component in astragali radix (AR), has become a research focus. However, the elaborate mechanisms of calycosin treating AIC remain to be unrevealed.

**Aim of the study:**

To explore the effects of CA on AIC through multiple dimensions concerning network pharmacology, molecular docking, and experimental evaluations.

**Methods:**

The study evaluated calycosin's potential targets and mechanisms for treating AIC using network pharmacology and molecular docking. The candidate genes/targets of CA and AIC were screened using the online-available database. Protein-protein interactions (PPI) between the common targets were constructed using the STRING platform, and the results were then visualized using Cytoscape. Molecular docking was used to evaluate the strength of the binding force between CA and the common targets. The possible pharmacological mechanisms of CA were explained by pathway enrichment and GSEA. Subsequently, the candidate targets were identified *in vitro* experiments.

**Results:**

Network pharmacology effectively discovered the CA's multitarget intervention in AIC, including TNF, ABCC1, TOP2A, ABCB1, and XDH. CA binds to the ATP-binding cassette subfamily B member 1(ABCB1) had the highest binding energy (−7.5 kcal/mol) according to the molecular docking analysis and was selected and visualized for subsequent analysis. In vitro experiments showed that ABCB1 exhibited significant time-curve changes under different doses of doxorubicin (DOX) compared with DMSO control experiments. The anti-AIC pharmacological mechanism of CA were revealed by highlighting the biological processes of oxidative stress (OR) and inflammation.

**Conclusions:**

We employed a practicable bioinformatics method to connect network and molecular docking to determine the calycosin's therapeutic mechanism against AIC and identified some bioinformatics results in *in vitro* experiments. The results presented show that CA may represent an encouraging treatment for AIC.

## Introduction

Anthracycline antineoplastic agents (ANTs), such as doxorubicin (DOX) and epirubicin, have been perceived as one of the most effective anticancer drugs widely employed in solid tumors and hematologic malignancies; however, its dose-dependent cardiotoxicity, which affects the progress of subsequent treatment to a great extent (1–4), limits its clinical practice in a great measure. ANTs-induced cardiotoxicity (AIC), described by heart function or structure changes, can lead to a progressive cardiomyopathy and ultimately lead to heart failure (5). AIC research mainly focuses on oxidative stress (6), mitochondrial dysfunction (7), inflammation (8), autophagy, apoptosis (9, 10), pyroptosis (11), ferroptosis (12), and altered Ca^2+^ homeostasis (13). The role of oxidative stress, which represents an imbalance between the production of ROS and antioxidant protection effect (14), in the development and progression of AIC is an essential academic area that is currently well-studied (6, 10, 15, 16). Although multiple drugs have been demonstrated to mitigate the adverse effects of DOX (17, 18), dexrazoxane is the only prescription authorized by the U.S. Food and Drug Administration for the treatment of DOX induced cardiotoxicity (19). However, clinics restrict it due to serious side effects that increase the risk of myelodysplastic syndrome and acute myeloid leukemia. Thus, to better prevent and manage this challenge, there is a pressing need for more effective therapeutic agents and strategies.

**Figure 1:**
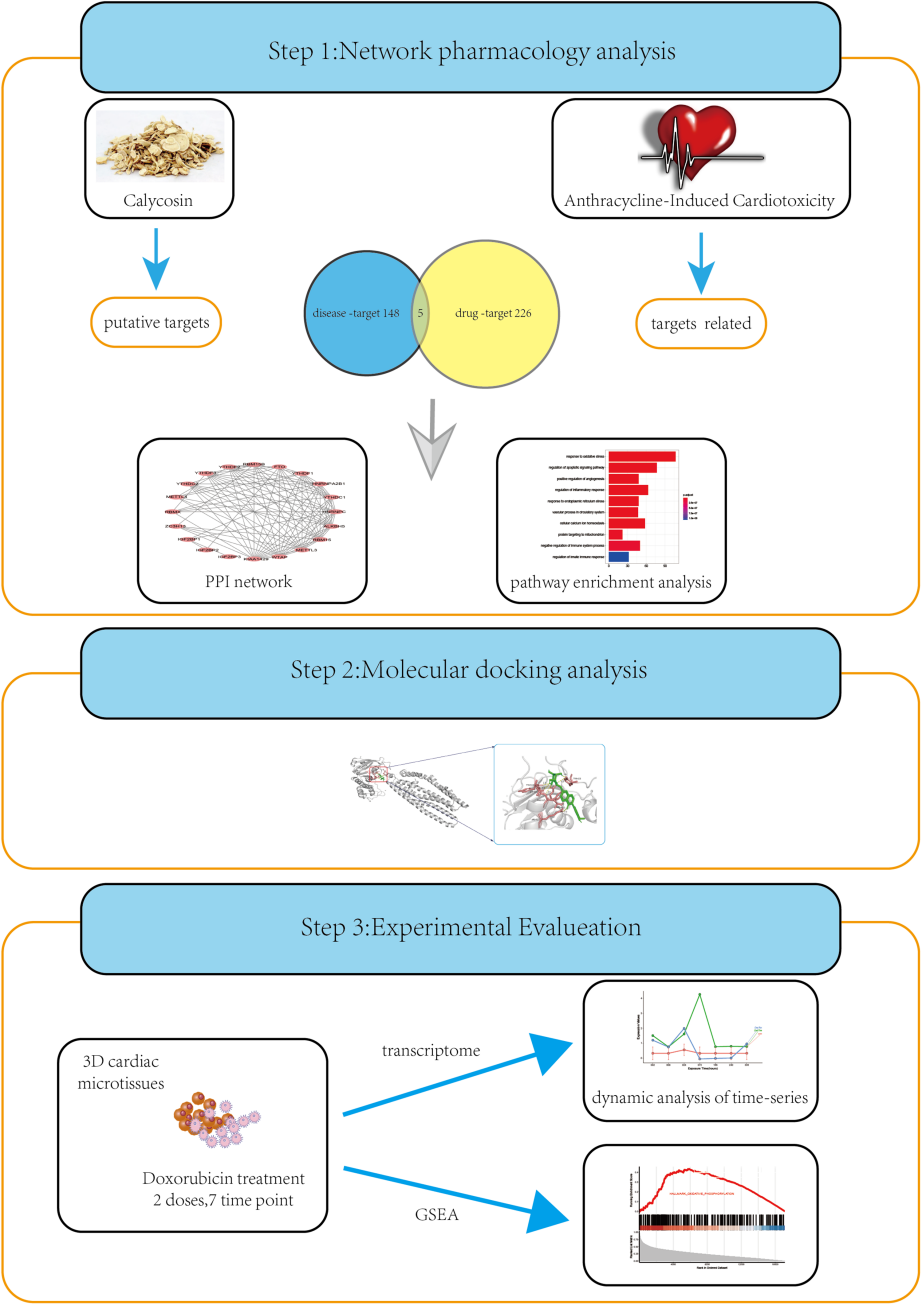
A strategy combining network pharmacology, molecular docking, and experimental evaluations to highlight the core targets and biological functions of calycosin treating AIC. The common targets of CA treating AIC and AIC were screened using network pharmacology. The Molecular docking was the used to evaluate the candidate targets and the candidate targets were subsequently identified *in vitro* experiments.The possible pharmacological mechanisms of CA were explained by pathway enrichment and GSEA.

Natural compounds have a significant role in oncology drug research as well as drug discovery. Researchers have discovered that certain components in traditional Chinese medicine may counteract the harmful effects of chemoradiotherapy on the heart while maintaining the treatment's ability to fight tumors (17). This could significantly raise the survival rates and quality of life for people receiving this treatment. For example, calycosin (C16H12O5, CA; Figure 1), an isoflavonoid that is the major active component present in Radix astragalithe, is growing into a highly valued herb used in traditional Chinese medicine to treat cardiovascular disease (18, 19). CA has been interpreted in numerous bioactivities, including anti-inflammatory, antioxidative, antiapoptotic, anticancer, immunomodulating, and cardiovascular protection (20–26). Yet, there have been few studies addressing the role of calycosin in drug-induced myocardial damage. Following CA therapy in vivo and in vitro, the DOX-induced damage to cardiomyocytes was significantly reduced. Furthermore, the reduction of ROS generation, inflammation, autophagy, apoptosis, and pyroptosis could be responsible for the cardioprotective properties of CA. By preventing the expression of pro-inflammatory chemicals, CA serves to protect cardiomyocytes from DOX-induced injury. This has been mainly shown by a reduction in NLRP3 and systemic inflammation. In regards to one study, CA can reduce DOX-induced cardiotoxicity while also lowering cell apoptosis and inhibiting oxidative stress both *in vivo* and *in vitro* by modulating the Sirt1-NLRP3 pathway (20, 27). Through the NLRP3-caspase-1-GSDMD pathway *in vitro* and *in vivo* experiments, CA reduces doxorubicin-induced ROS, mitochondrial damage, and cardiomyocyte pyroptosis, a crucial immune response that is linked to pro-inflammatory effects (26). Furthermore, it has been reported that CA modifies Atg7-related autophagy to protect against DOX-induced cardiotoxicity (28). Calycosin improves embryo viability and reduces pericardial edema and morphological changes induced by doxorubicin in a zebrafish model (28). However, the findings are still debatable. It is known that inflammation plays a role in DOX-induced cardiotoxicity. However, inflammation is even worse once cardiotoxicity manifests (29). Preliminary evidence suggests that CA's cardioprotective action is closely related to its claimed bioactivities, although some of this correlation remains controversial. There has been no systematic investigation into the effects and pharmacological actions of calycosin against AIC.

Therefore, we proposed the notion that the cotreatment of CA with chemotherapeutic drugs would play an impact boosting and toxicity decreasing role, taking use of the aforementioned features. The cardioprotective effects and potential mechanisms of CA on doxorubicin-DOX-induced cardiotoxicity merit further research given that numerous active ingredients in traditional Chinese medicine exhibit noteworthy protective effects on DOX-induced cardiotoxicity through interactions with multiple targets.

Network pharmacology, analyzing the molecular association between drugs and treatment targets and revealing the systematic pharmacological mechanisms of drugs from a system-level perspective using a network, was first proposed in 2007 (30). Drug development will become more cost-effective owing to network pharmacology-based drug research and the burgeoning fields of bioinformatics and integrated pharmacology. By modelling the interactions between ligands and receptors, molecular docking can also forecast binding capability and conformation accurately (31). The current network pharmacology and molecular docking-based approach identified the potential targets, functions, and signaling pathways of calycosin against AIC. It is presumed that functional regulation of these ideal biological processes and signaling pathways by calycosin could contribute to an anti-AIC effect. Additionally, calycosin’s principal pharmacological biotargets have been assessed.

## Materials and methods

### Medical ethics

This study involved bioinformatics analysis only and thus did not require medical ethics approval. The study followed the Declaration of Helsinki (as revised in 2013).

### Collection of all targets of calycosin and AIC

The molecular structure of calycosin was downloaded from PubChem (https://pubchem.ncbi.nlm.nih.gov/) (32). The therapeutic targets of calycosin were collected in the PubMed database, Traditional Chinese Medicine Systems Pharmacology Database and Analysis Platform (TCMSP; https://old.tcmsp-e.com/tcmsp.php), Chemical-Protein Interaction Networks (STITCH; http://stitch.embl.de/), Swiss Target Prediction (https://www.swisstargetprediction.ch/), SuperPred web server (https://prediction.charite.de/), Bioinformatics Analysis Tool for Molecular mechANism of Traditional Chinese Medicine (BATMANTCM; http://bionet.ncpsb.org.cn/batman-tcm/), and ChemMapper (http://www.lilab-ecust.cn/chemmapper). All the datebase used “calycosin” or the molecular structure of calycosin as search keywords, and only collected species limited as “Homo sapiens”.

The ACT-related targets were identified using the query term “anthracycline, cardiotoxicity” from PharmGkb database (https://www.pharmgkb.org/), OMIM database (https://www.omim.org/) (33), GeneCards database (https://www.genecards.org/) (34), The genetic association database (GAD) database (https://geneticassociationdb.nih.gov/) and DISGNET database (https://www.disgenet.org/home/), which offers information about disease targets.

### Obtaining intersection target genes of calycosin and AIC

After the genes/targets of calycosin and AIC had been mapped by using R language (R version 4.2.1), the overlapping targets determined by the Venn diagram were selected as the predictive targets of calycosin against AIC.

### Constructing protein-protein interaction network

To clarify the interaction of the genes targeted for therapy, the acquirable targets of calycosin and AIC were connected methodologically to create a calycosin-based anti-AIC network through a tool of the Search Tool for the Retrieval of Interacting Genes/Proteins database (STRING, https://string-db.org/) (35). The common targets obtained by Venn analysis were input into the STRING platform. The species were set to “Homo sapiens,” and the minimum required interaction score was “high confidence.” Furthermore, these screened targets were further analyzed after creating a protein-protein interaction (PPI) network based on the Cytoscape tool. Previous studies have shown that proteins exert biological activities through protein-protein interactions (PPI).

### Functional process and molecular pathway of calycosin for AIC

To identify underlying functional processes and signaling pathways of calycosin treating AIC, the Gene Ontology (GO) pathway enrichment analysis was carried out using the R packages ClusterProfiler (version 4.4.4) and AnnotationHub (version 1.60.2). The parameters were as follows: the minimum number of genes was 2, the maximum number of genes was 5,000, and the *P* value was <0.05. Gene set enrichment analysis (GSEA) was performed using ClusterProfiler (version 4.4.4) to quantify the associations between the target gene and each gene set. The genes were ranked based on the strength of the association between the target gene and the RNA sequencing data, quantified by Pearson's correlation coefficients. Gene sets with a false discovery rate (FDR) <0.25 were considered significant.

### Molecular docking study for predicted target validation

In order to investigate the the binding affinities between calycosin molecule and its predicted proteins, molecular docking was performed. Molecular docking using Autodock Vina 1.5.6 software (http://autodock.scripps.edu/) is generally divided into four steps: preparation of protein, preparation of ligand and the standard inhibitor, docking and analysis of results.

#### Ligand and protein preparation for autodock vina

The protein structure was downloaded from Protein Data Bank (https://www.rcsb.org/), while the crystal structure of CA and the standard inhibitor were downloaded from PubChem databases (https://pubchem.ncbi.nlm.nih.gov/) (39), then converted to three-dimensional (3D) structure and energy minimized using OpenBabel 2.4.1. The ligands and proteins were prepared through Autodock Vina, including removal of water molecules, addition with hydrogens and so on. The prepared pdb files of the ligand and proteins were then converted into the pdbqt format by assigning charges using Autodock Vina.

#### Docking and analysis of results

The protein file to be docked was selected in macromolecular input of Autodock Vina, and the ligand file to be docked was selected in ligand input. Secondly, the active pocket (the position of the original ligand in the protein crystal, including all residues around the original ligand) on protein structure was determined by GetBox-PyMOL-Plugin (https://github.com/MengwuXiao/GetBox-PyMOL-Plugin). The values of the utilized parameters in Autodock Vina software were presented in Supplementary Table S1.

The docking results showed 9 different conformations, ranked according to the values of predicted binding-energy value, and higher binding energy indicate a greater possibility of the the drug binding to the protein. The docking models with the highest binding-energy were selected and visualized for subsequent analysis. The PyMOL Molecular Graphics System,version 2.5.5 (https://pymol.org/2/) was then used to prepare the figures to visualize the drug-protein interactions.

### Identification of the target genes

The human cardiac microtissues, containing 4,000 iPS-derived human cardiomyocytes from a female Caucasian donor and 1,000 cardiac fibroblasts from a male Caucasian donor, were exposed to DOX for 2 weeks. The cardiac microtissues were subjected to incubation with either a therapeutic or toxic dosage. The therapeutic dosage is equivalent to the clinical dosage, while the toxic dosage is based on the IC20 value determined through ATP production (cell viability) after a 7-day exposure (36). The RNA expression data were harvested after 2, 8, 24, 72, 168, 240, and 336 h of DOX exposure with 3 replicates per dose. MaSigPro (37) was used to utilize the time-series data generated by each gene to estimate its temporal response and the importance of the deviation of the treatment-time curve from the respective control curve. The above information is publicly accessible at the Hepatic and Cardiac Toxicity Systems modeling project funded by the European Union Seventh Framework Programme (FP7/2007-2013) (38).

## Results

### Biological targets and PPI network of calycosin treating AIC

The flowchart of this article is shown in Figure 1.

**Figure 2:**
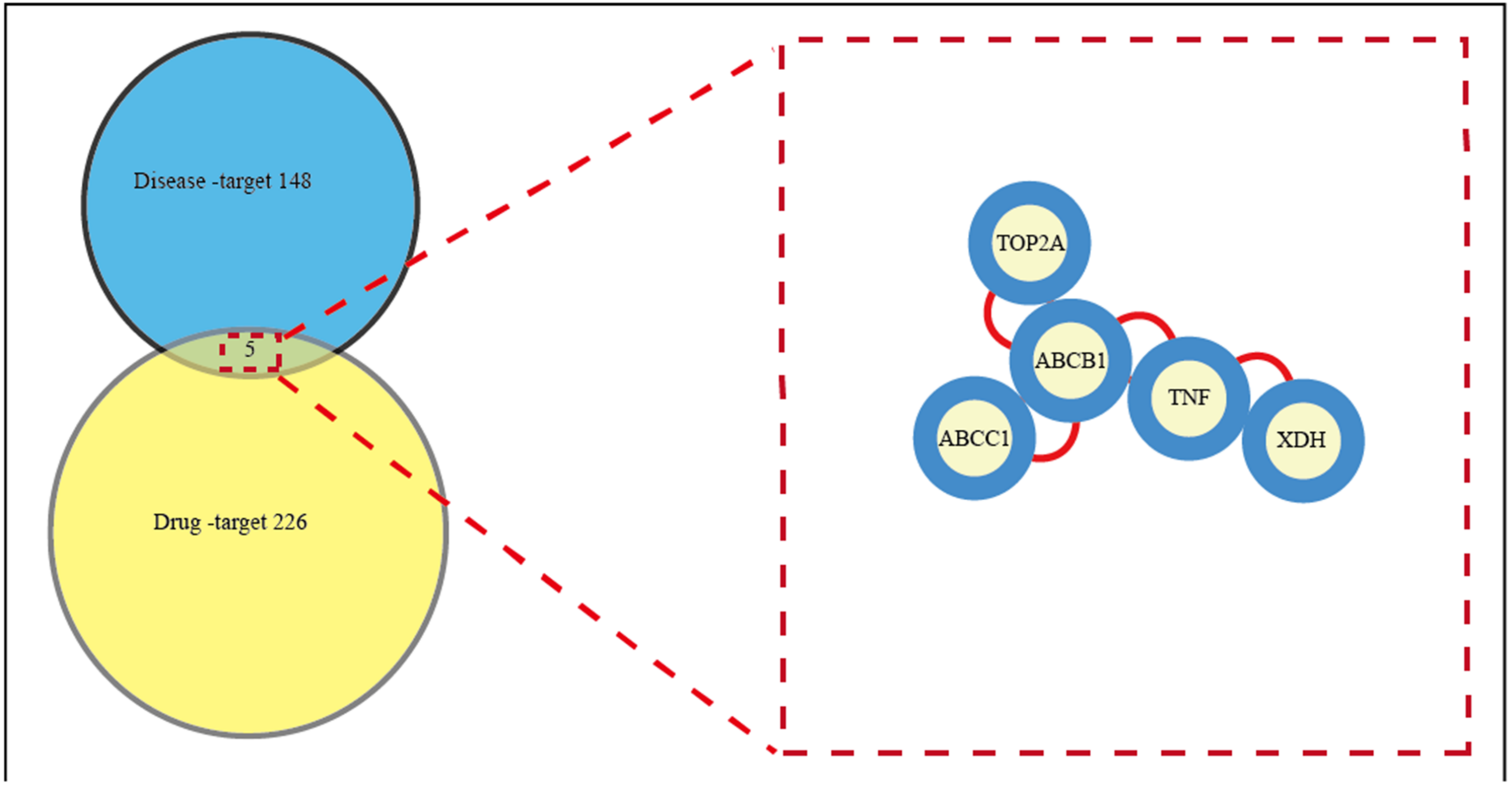
All candidate biotargets of calycosin and AIC were identified using network pharmacology, and then a PPI network from these biotargets was represented. This network effectively displays the CA's multitarget intervention in AIC. Left: Venn diagram of targets shared by CA and AIC. 226 drug-related targets of CA and 148 AIC disease-related targets were gathered. Right: Common targets screening in the PPI network. Drug-related and disease-related targets were intersected, and 5 central genes/targets of calycosin for AIC were displayed. PPI, protein-protein interaction.

The study evaluated calycosin's potential targets and mechanisms for treating AIC using network pharmacology and molecular docking. In the present study, 226 drug-related targets of CA and 148 AIC disease-related targets were gathered. Drug-related and disease-related targets were intersected, and 5 central genes/targets of calycosin for AIC were displayed in a Venn diagram (Figure 2). And the central targets are detailed as TNF, ABCC1, TOP2A, ABCB1, and XDH. These interceptive targets in the Venn diagram were used to further build an optimal PPI network in order to investigate the protein interactions and putative mechanism of CA in treating AIC. This network effectively displays the CA's multitarget intervention in AIC.

### Revelation of biological functions and pathways from the central targets

**Figure 3:**
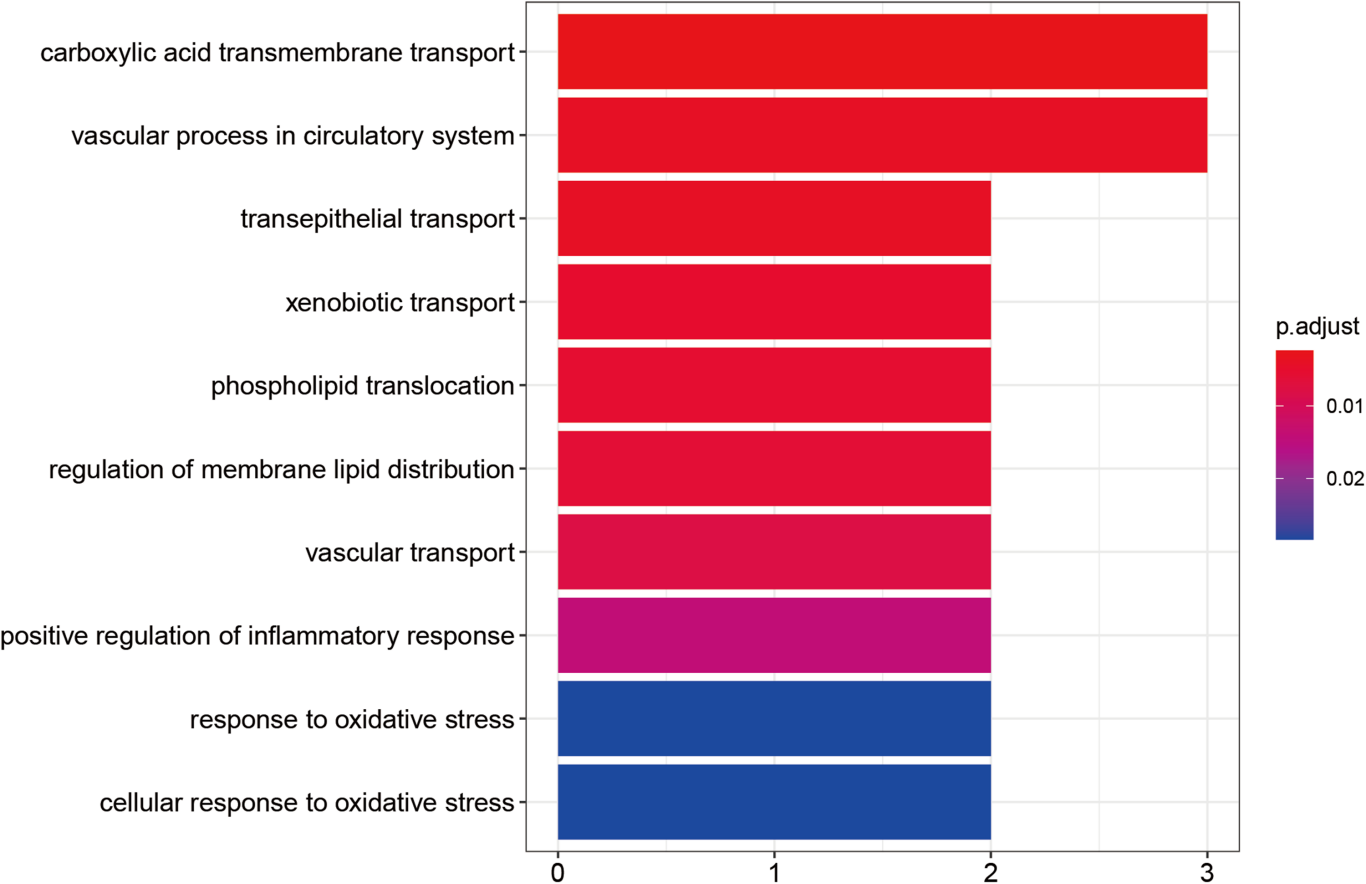
Calycosin's essential biological and functional roles in combating AIC by gene ontology enrichment analysis. The color represents the size of the *P* value. Redder colors indicate smaller *P* values and closer relationships. Bluer colors indicate larger *P* values and more distant relationships.

All central targets of calycosin for anti-AIC were enriched to display molecular pathways. As a result, the major targets could be divided into various functional categories by Gene Ontology enrichment analysis, including positive regulation of inflammatory response, response to oxidative stress, and cellular response to oxidative stress (Figure 3).

### Molecular docking

**Figure 4:**
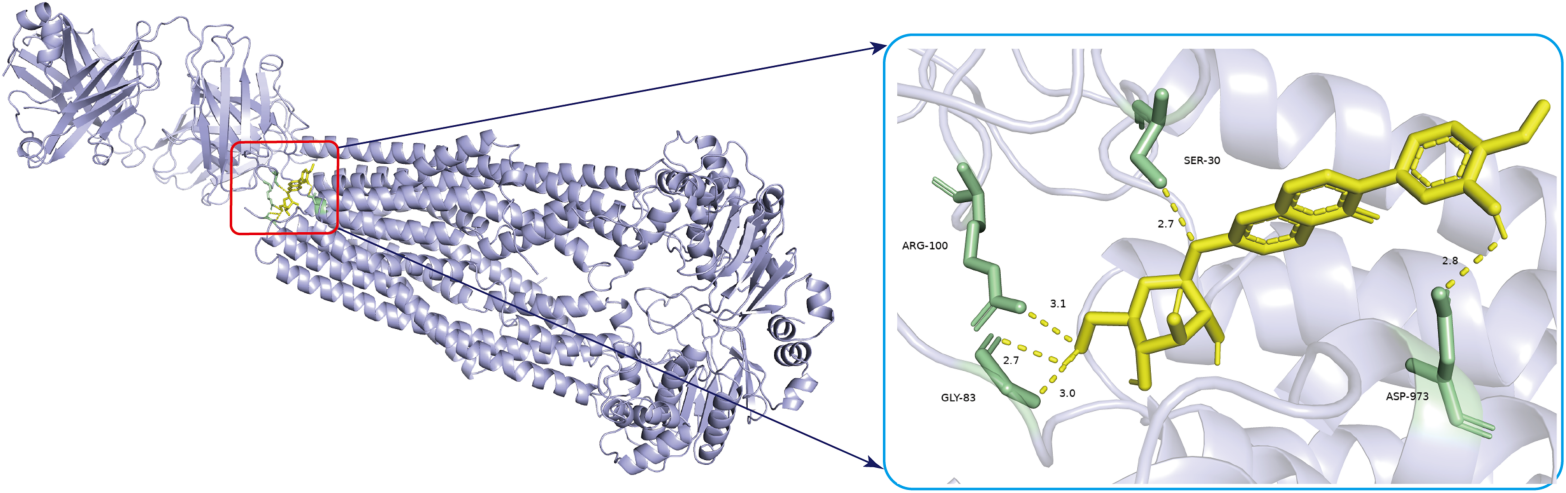
The 3D diagrams of calycosin and its binding to ABCB1 are shown. The yellow image represents CA and yellow hydrogen bonds connect the binding sites. The length of the hydrogen bonds is indicated next to the bond. The protein target that binds to CA is grey, and the amino acid residues is green.

Molecular docking analysis was used to evaluate the binding energies of the CA for all central targets. The results indicated that CA (PubChem CID 5280448) binds to ABCB1 (PDB ID, 7A69; resolution, 3.2 Å) (39) had the highest binding energy (−7.5 kcal/mol). According to broad consensus, a docking score of less than 0 kcal/mol means that the component can spontaneously interact to the target, a score of less than −4.25 kcal/mol means that the docking affinity is good, and a score of less than −7 kcal/mol means that the component has strong docking affinity. The standard inhibitor verapamil (PubChem CID 2520) used to compare the docking efficacy, showed −6.0 kcal/mole binding efficacy. The 3D binding diagram, hydrogen bonding diagram and the amino acid residues were displayed in Supplementary Figure S1. The values of binding energies of the CA for other central targets and the utilized parameters in Autodock Vina software were presented in Supplementary Tables S1,S2. Figure 4 displays the 3D binding diagram and hydrogen bonding diagram, along with detailed information on molecular docking. It can be seen that CA has developed many hydrogen bonds with the amino acid residues ARG100, SER-30, GLY-83and ASP-973.

### ABCB1 is involved in AIC

**Figure 5:**
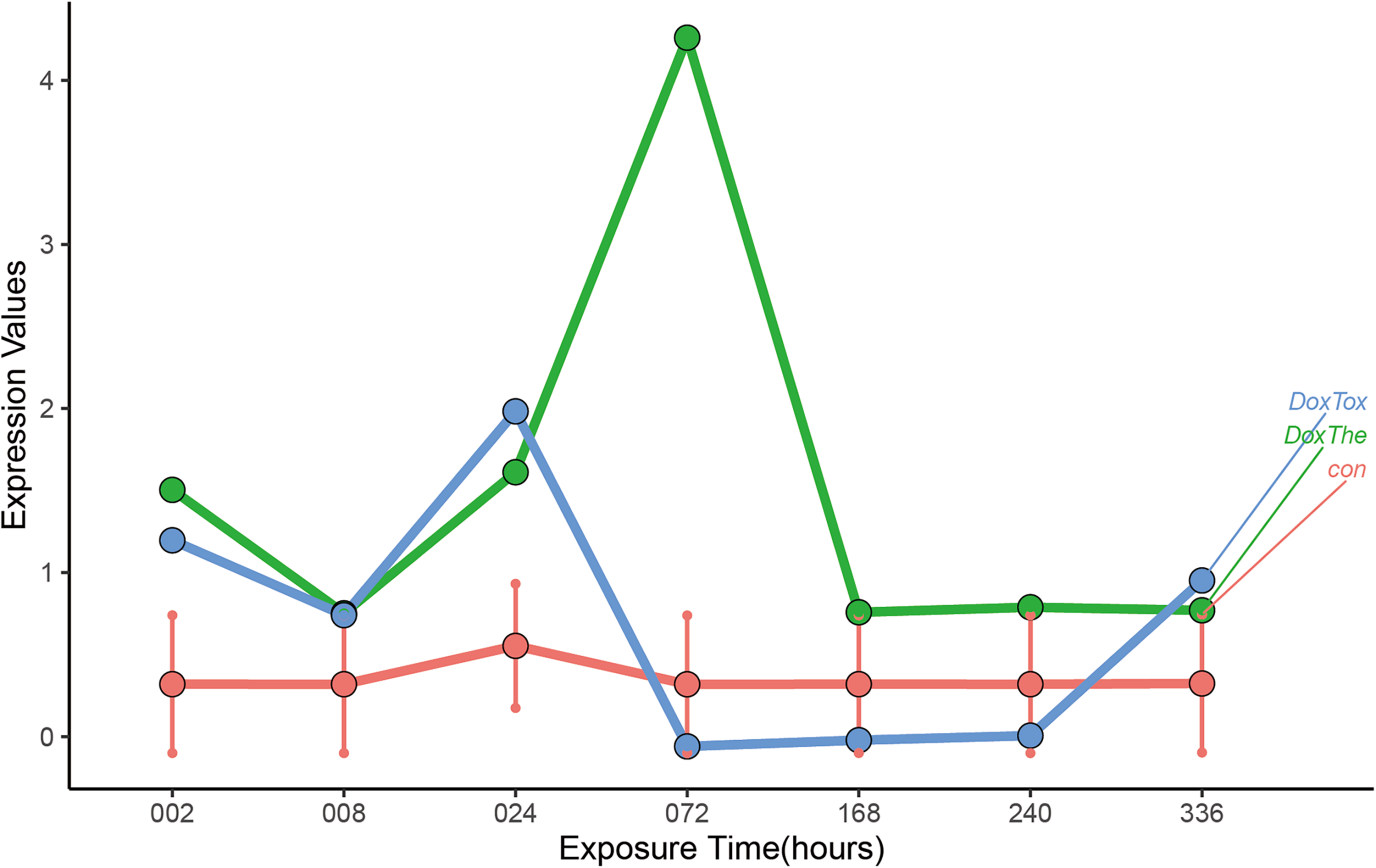
The dynamic changes of ABCB1 *in vitro* human 3D cardiac microtissues subjected to either a therapeutic dose or a toxic dose of DOX. The: therapeutic dose, Tox: toxic dose; 002, 008, 024, 072, 168, 240, 336 are corresponding exposure periods in hours; Dox: doxorubicin.

To identify the target genes, human 3D cardiac microtissues subjected to either a therapeutic dose or a toxic dose of DOX were evaluated. By comparing the DOX treatment time profiles with control profiles from time-matched DMSO-treated microtissues, we found that ABCB1 displayed significant changes in time profiles with different doses compared to the DMSO control experiments (Figure 5). Gene set enrichment analysis (GSEA) was used to identify various pathways with ABCB1, and ABCB1 was most significantly connected with HALLMARK_OXIDATIVE_PHOSPHORYLATION, HALLMARK_MYOGENESIS, HALLMARK_PEROXISOME (Supplementary Figure S2).

## Discussion

Anthracycline chemotherapeutics, addressed by doxorubicin, play an important role in treating various cancers. Unfortunately, side effects such as cardiotoxicity have severely confined the clinical application of these drugs. AIC's precise mechanism remains unclear. Calycosin, as a multi-target anti-tumor drug, has been widely studied. However, the effectiveness of CA for treating AIC and the underlying molecular mechanism has not yet been clarified. Hopkins first introduced network pharmacology, which has been applied to investigate pharmacological effects or show the synergism function mechanism of multiple component drugs (40). The powerful methodology of joining network pharmacology with molecular docking might assist targets and pathways for calycosin in AIC treatment. Based on the network pharmacology integrated molecular docking, we concluded that the main target of CA in treating AIC might be ABCB1. Additionally, the biological mechanisms of oxidative stress (OR) and inflammation were emphasized, illuminating the pharmacological underpinnings of calycosin's anti-AIC effects.

A total of 5 cross-targets of CA and AIC were obtained by constructing a Venn diagram, and the leading core target was found to be ABCB1 through molecular docking analysis. ABCB1 exhibited the strongest binding affinity with calycosin, suggesting that this gene could serve as a powerful pharmacological target to combat AIC. ABCB1(P-gp, MDR1) (41) is one of the membrane transporters expressed at high levels in cardiomyocytes and cardiac capillary endothelial cells of the mammalian heart. It is responsible for DOX cell influx and efflux and regulates both intra- and extracellular concentrations and bioavailability of the drug and its metabolites. Recent research has revealed that tissue-specific transporter expression is involved in local drug accumulation and drug-drug interactions and that perturbations to these transporters’ functions could determine the vulnerability an individual is to drug-induced toxicity. ABCB1 typically works to extrude drugs or toxic xenobiotic substances from cells or tissues and has been specifically studied with cardiotoxicity (42, 43). It is speculated that ABCB1 transports anthracyclines extracellularly and reduces their intracellular accumulation, resulting in the resistance of cancer cells and the protection of healthy cells. Numerous animal models have demonstrated that inhibiting endogenous ABCB1 increases the risk of doxorubicin-induced cardiotoxicity (44, 45). In the hearts of Abcb1a_/_ mice, despite only a 1.2-fold increase in plasma levels compared to wild-type mice, the retention of doxorubicin and its main metabolite, doxorubicin, is substantially prolonged (45). In the hearts of HSF-1/_ mice heart, the increased expression of ABCB1 enhanced the extrusion of doxorubicin and diminished left ventricular dysfunction (46). Notwithstanding, clinical proof of this proposition remains controversial (47). It is also worth pointing out that ABCB1 transports many cancer drugs very efficiently but still causes shifting levels of cardiotoxicity in patients, an observation that appears to be conflicting with ABCB1 serving a vital protective capability. The link between genetic polymorphisms in the ABCB1 gene and cardiotoxicity was identified (43). The variant allele of ABCB1 3435C>T (rs1045642) had an additive protective effect against cardiotoxicity in breast cancer patients treated with anthracyclines (43), and the results remained nominally significant after adjustment for clinical covariates. According to a meta-analysis, ABCB1 rs1045642 is associated with a lower risk of developing AIC (48). Only a little amount of research was conducted on the targets relating calycosin against AIC.

Our results showed that calycosin ameliorates AIC involved with oxidative stress (OR) and inflammation, which were representatively validated *in vivo* and *in vitro*. The most significant molecular mechanisms for the pathophysiology of chemoradiotherapy cardiotoxicity are OR and inflammation. Oxidative stress is brought on by the breakdown of redox equilibrium, which is manifested by an increase in ROS and a decrease in antioxidant enzymes (15, 16). Inflammation is a key component of OR-related cardiotoxicity caused by chemotherapy drugs. Interleukin-1 (IL-1) and tumor necrosis factor (TNF) are two inflammatory factors that DOX increases in the heart, prompting inflammatory and immunological responses and impairing cardiomyocytes (49). NLRP3-related signaling pathways regulate the release of inflammatory factors. The NLRP3 inflammasome is a key mediator of the innate immune system, mediating caspase-1 activation and secretion of the proinflammatory cytokines IL-1β/IL-18 (50). NLRP3 inflammatory vesicles are activated by DOX-induced ROS (11), and calycosin may reduce inflammation and OR by boosting the levels of NLRP3 and associated proteins in cells and mouse hearts (20, 51). Calycosin minimized AIC and increased the viability of rat cardiomyocytes by blocking the NLRP3/caspase-1/GSDMD pathway (26, 52). According to a similar study, CA can reduce DOX-induced cardiotoxicity via controlling the Sirt1-NLRP3 pathway, which also reduces oxidative stress both *in vivo* and *in vitro* (20).

This study also has some limitations. Calycosin is a monomer molecule with a definitive chemical structure characterized by low toxicity and a range of biological effects (53). This makes it more conducive to research and applications in pharmacology research. Calycosin and its derivatives have good cardiovascular protective potential. However, existing studies have only addressed the role of calycosin in ischemic disease, myocardial hypertrophy, and viral myocarditis. Fewer studies have examined pathological circumstances such as drug-induced myocardial damage. Robust patient cohorts in clinical trials are needed to confirm the efficacy of calycosin. Furthermore, drug targets involved in cardiotoxicity need larger samples to expand. Thirdly, although the key targets and pathways can be screened in the research, further phytochemical and pharmacological research is needed to determine the exact mechanism. Significantly, calycosin may influence the corresponding signaling pathways under different pathological conditions.

The study established a network pharmacology-integrated molecular docking strategy, and through partial *in vitro* verification, highlighted the therapeutic targets, functional processes, and molecular mechanisms of calycosin for AIC through partial verification *in vitro*. Based on the network pharmacology integrated molecular docking, we concluded that the main target of CA in treating AIC might be ABCB1. Additionally, the biological mechanisms of oxidative stress (OR) and inflammation were emphasized, illuminating the pharmacological underpinnings of calycosin's anti-AIC effects. Additional validated experiments will be performed in a preclinical research during subsequent studies. The foundation for the clinical application is laid by this study's effective combination technique for systematically elucidating medication therapeutic mechanisms.

## Data Availability

Publicly available datasets were analyzed in this study. This data can be found here: Hepatic and Cardiac Toxicity Systems modeling project funded by the European Union Seventh Framework Programme (FP7/2007-2013). Further inquiries can be directed to the corresponding author.

## Supplementary material

Supplementary Figure S1 The 3D diagrams of the standard inhibitor and its binding to ABCB1.

Supplementary Figure S2 The ABCB1 associated pathways based the GSEA analysis.

Supplementary Table S1 The utilized parameters in Autodock Vina software.

Supplementary Table S2 Docking score of CA and the central targets.
