# Supplementary figures and images for: Exploring the effects of calycosin on anthracycline-induced cardiotoxicity: a network pharmacology, molecular docking, and experimental study
Source: Front Cardiovasc Med. 2024 Mar 21;11:1286620. doi: 10.3389/fcvm.2024.1286620 (PMC10991710; doi:10.3389/fcvm.2024.1286620)

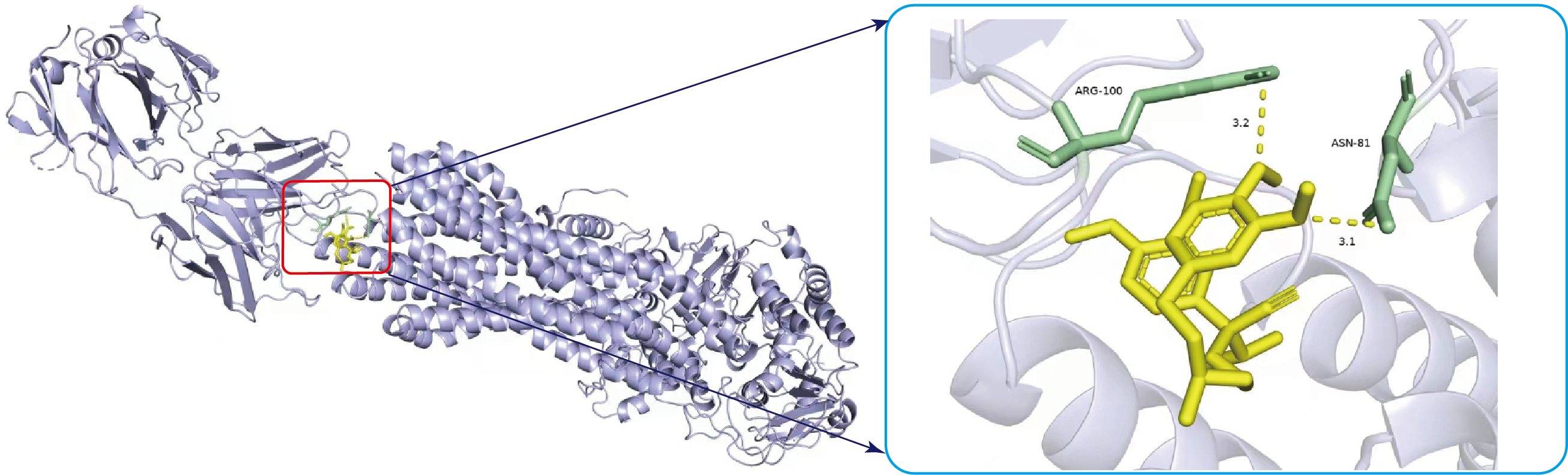

Supplement: Supplementary Figure S1 — The 3D diagrams of the standard inhibitor and its binding to ABCB1. [file Image1.tif]

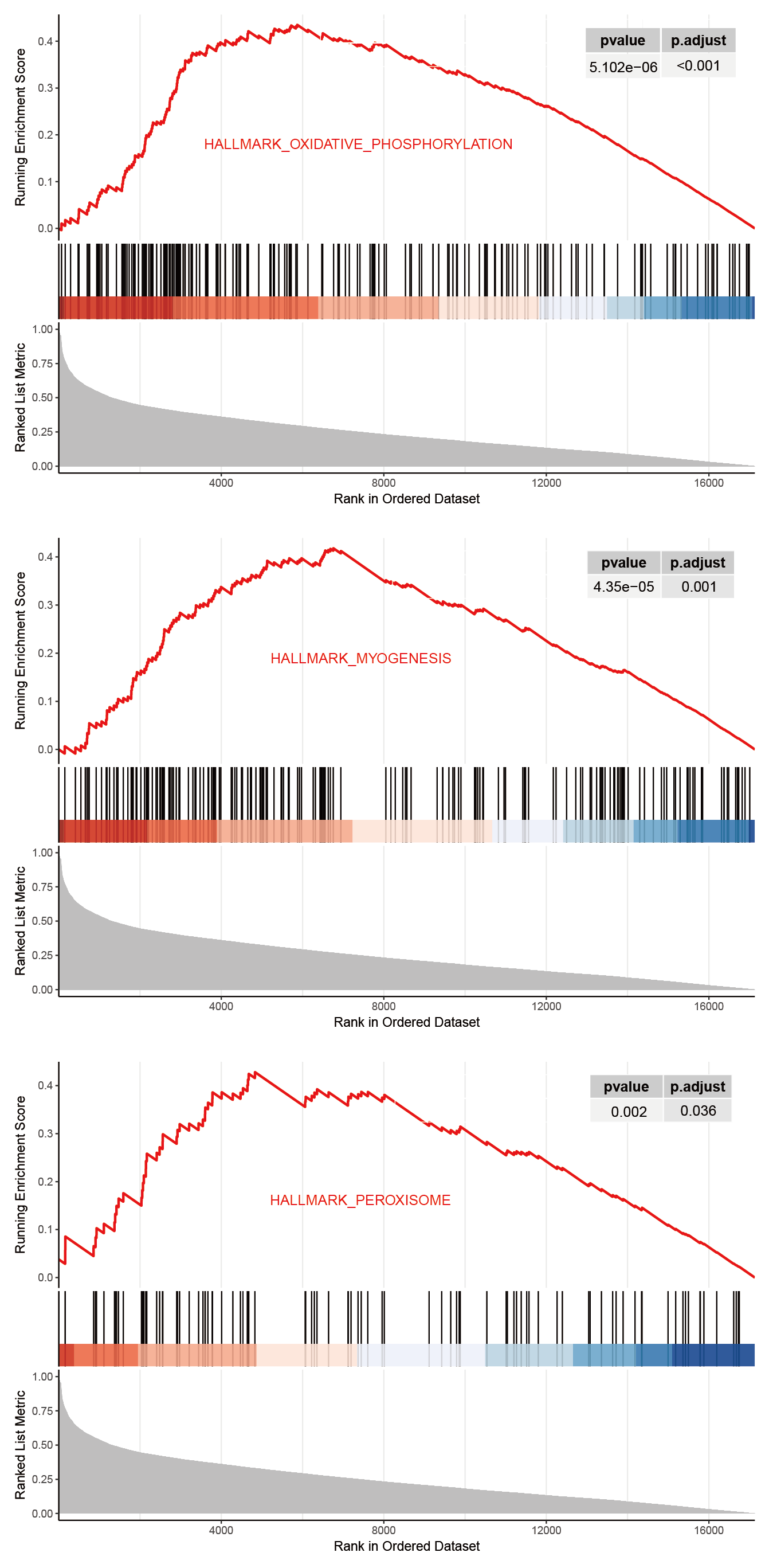

Supplement: Supplementary Figure S2 — The ABCB1 associated pathways based the GSEA analysis. [file Image2.tif]
